# Differential intracellular fate of *Burkholderia pseudomallei *844 and *Burkholderia thailandensis *UE5 in human monocyte-derived dendritic cells and macrophages

**DOI:** 10.1186/1471-2172-10-20

**Published:** 2009-04-27

**Authors:** Jaruek Charoensap, Pongsak Utaisincharoen, Anneke Engering, Stitaya Sirisinha

**Affiliations:** 1 Department of Microbiology, Faculty of Science, Mahidol University, Bangkok, Thailand; 2 Department of Molecular Cell Biology and Immunology, VU Medical Center, Amsterdam, The Netherlands

## Abstract

**Background:**

*Burkholderia pseudomallei *(*Bp*) is a category B biothreat organism that causes a potentially fatal disease in humans and animals, namely melioidosis. *Burkholderia thailandensis *(*Bt*) is another naturally occurring species that is very closely related to *Bp*. However, despite this closely related genotype, *Bt *is considered avirulent as it does not cause the disease. In the present study, we compared the growth kinetics of *B. pseudomallei *strain 844 (*Bp*-844) in human monocyte-derived dendritic cells (MoDCs) and macrophages (Mφs), as well as its ability to stimulate host cell responses with those of *B. thailandensis *strain UE5 (*Bt*-UE5).

**Results:**

Primary human MoDCs and Mφs were infected with *Bp*-844 and its intracellular growth kinetics and ability to induce host cell responses were evaluated. The results were compared with those obtained using the *Bt*-UE5. In human MoDCs, both bacteria were similar in respect to their ability to survive and replicate intracellularly, induce upregulation of costimulatory molecules and cytokines and bias T helper cell differentiation toward a Th1 phenotype. By contrast, the two bacteria exhibited different growth kinetics in human Mφs, where the intracellular growth of *Bt*-UE5, but not *Bp*-844, was significantly suppressed. Moreover, the ability of Mφs to kill *Bp*-844 was markedly enhanced following stimulation with IFN-γ.

**Conclusion:**

The data presented showed that while both strains were similar in their ability to survive and replicate in human MoDCs, only *Bp*-844 could readily replicate in human Mφs. Both bacteria induced similar host cellular responses, particularly with regard to their ability to bias T cell differentiation toward a Th1 phenotype.

## Background

Melioidosis is a serious infectious disease caused by *Burkholderia pseudomallei *(*Bp*), a gram-negative bacterium that is classified as a category B bioterrorism agent by Centers for Disease Control and Prevention [1]. It is found in soil and water and is endemic in the region between 20°N and 20°S of the equator including Southeast Asian countries and northern Australia [2]. This soil-saprophyte is a facultative intracellular organism that can multiply readily in murine phagocytic and non-phagocytic cell lines [3]. It is the causative agent of the potentially fatal disease, meloidiosis, which occurs in both humans and animals. Human melioidosis has a broad clinical spectrum ranging from a seropositive subclinical condition to an acute fatal septicemia [2].

*B. thailandensis *(*Bt*) is a non-pathogenic environmental saprophyte that was formerly considered to be an avirulent biotype of *Bp *[4]. It is genetically and phenotypically very similar to *Bp *but is markedly less virulent in both human and animal models. For example, *Bp *is at least 10^6^-fold more virulent than *Bt *in BALB/c mice and so far none of the 1,200 clinical isolates reported have displayed the *Bt *phenotype [4]. *Bt *is usually present together with *Bp *in the regions where melioidosis is endemic, e.g., northeast Thailand [5]. In order to further delineate the pathogenesis mechanism(s) of *Bp *in humans, which remain largely unresolved, the *in vitro *interactions of *Bp *strain 844 and *Bt *strain UE5 with primary human phagocytic cells were compared. We previously showed that *Bp *adhered to and invaded a human epithelial cell line more efficiently than *Bt *[6]. In the present study, we continued this line of study by comparing the intracellular survival and growth kinetics of *Bp*-844 and *Bt*-UE5 in primary human monocyte-derived dendritic cells (MoDCs) and human macrophages (Mφs) and the respective host cell responses with regard to costimulatory molecule expression, cytokine production and MoDC-driven allogeneic T cell differentiation.

## Results

### Intracellular survival and replication of *Bp*-844 and *Bt*-UE5 in human MoDCs and Mφs

**Figure 1:**
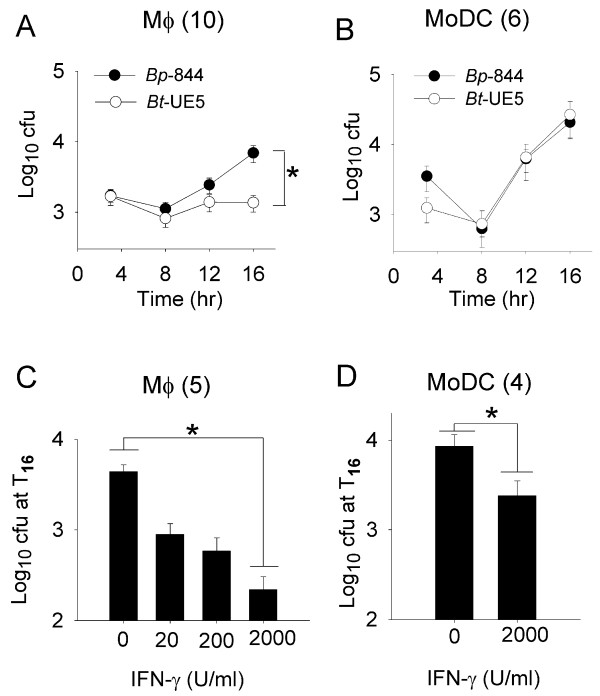
**Survival profiles of *Bp*-844 and *Bt*-UE5 in Mφs and MoDCs**. Immature (A and B) and IFN-γ activated (C and D) cells were exposed to *Bp*-844 and *Bt*-UE5 at a MOI of 1 and the mean numbers of intracellular bacteria determined up to 16 hr post-infection are shown. The error bars for each data point represent the standard error from the mean. Number in parenthesis indicates the number of donors in each experiment. * indicates a significant difference as analyzed with paired-samples *t*-test, with *P *< 0.01.

In order to study the intracellular survival and growth kinetics of both *Burkholderia *strains, primary human MoDCs and Mφs, cells were infected with *Bp*-844 and *Bt*-UE5 at a multiplicity of infection (MOI) of 1 and the number of viable intracellular bacteria was determined at 3, 8, 12 and 16 hr after infection (Figure 1A and 1B). Given the protocol used, the earliest time point where the number of intracellular bacteria could be determined was 3 hours post infection (T_3_). At this initial stage of infection, the mean level of intracellular *Bp*-844 in the MoDCs was higher than that in *Bt*-UE5 (Figure 1B), although the difference was not statistically significant (*P *= 0.106). As the infection progressed, the intracellular numbers of both bacteria, as measured at T_8_, declined compared with the 3-hr time point. At later time points (T_12 _and T_16_), however, both *Bp*-844 and *Bt*-UE5 multiplied exponentially inside MoDCs and exhibited similar kinetics until the experiment was terminated (T_16_), thus resulting in similar numbers of intracellular bacteria 16 hr after infection.

The results in Mφs were markedly different in that intracellular growth of *Bp*-844 was much better than that of *Bt*-UE5. While the numbers of intracellular bacteria were similar for both organisms in the early stages of growth (Figure 1A, T_3 _and T_8_), the number of intracellular *Bp*-844 was significantly higher at T_16 _(*P *< 0.01, paired-samples *t*-test). In fact, the growth of *Bt*-UE5 was completely halted between T_12 _and T_16_. The difference was more clear when comparing the intracellular growth rates of the two organisms in Mφs. *Bp*-844 grew with a doubling time of 3 hr 2 min in Mφs (T_8 _to T_16_) while that for *Bt*-UE5 could not be determined as the level of intracellular *Bt*-UE5 was relatively low and remained at the same level throughout the observation period. Moreover, the replication rate of both *Bp*-844 and *Bt*-UE5 during the exponential growth phase (T_8 _to T_16_) was higher inside MoDCs (1 hr 36 min) than that of *Bp*-844 in Mφs (3 hr 2 min). It should be mentioned that the intracellular growth rates of both organisms in both primary phagocytic cell cultures were considerably lower than those in the cell-free culture medium (40 min for *Bp*-844 and 37 min for *Bt*-UE5). In conclusion, whereas both *Burkholderia *stains survived and replicated equally well in MoDCs, the intracellular growth of *Bt*-UE5, but not *Bp*-844, was significantly suppressed in Mφs.

Pre-activation of Mφs with recombinant human IFN-γ for 16 hr prior to infection resulted in a reduction in the intracellular survival of *Bp*-844 in a dose-dependent manner, as judged from the number of intracellular bacteria determined 16 hr after the infection was started (Figure 1C). Although the intracellular survival was only significantly reduced when the IFN-γ was added to the Mφs at final concentration of 2000 U/ml (*P *< 0.01, paired-samples *t*-test), some reduction in survival, relative to the untreated control, could still be observed with IFN-γ pre-treatment at concentrations as low as 20 U/ml (Figure 1C). In MoDCs pre-treated with 2000 U/ml IFN-γ, a significant reduction (*P *= 0.049, paired-samples *t*-test, Figure 1D) in the intracellular level of *Bp*-844 was also observed, but the level of reduction was less than that noted in Mφs (Figure 1C). The effect of IFN-γ on bacterial survival was not the result of reduced bacterial uptake since both IFN-γ-treated and untreated cells displayed similar numbers of intracellular bacteria at the 3 hr post-infection time point (data not shown). Thus, this set of experiments showed that IFN-γ could stimulate Mφs, and to a lesser extent MoDCs, to acquire enhanced antibacterial killing capacity to reduce the intracellular survival and growth of *Bp*-844.

### Upregulation of costimulatory molecules, surface markers and cytokine production in *Bp*-844- and *Bt*-UE5-infected cells

**Figure 2:**
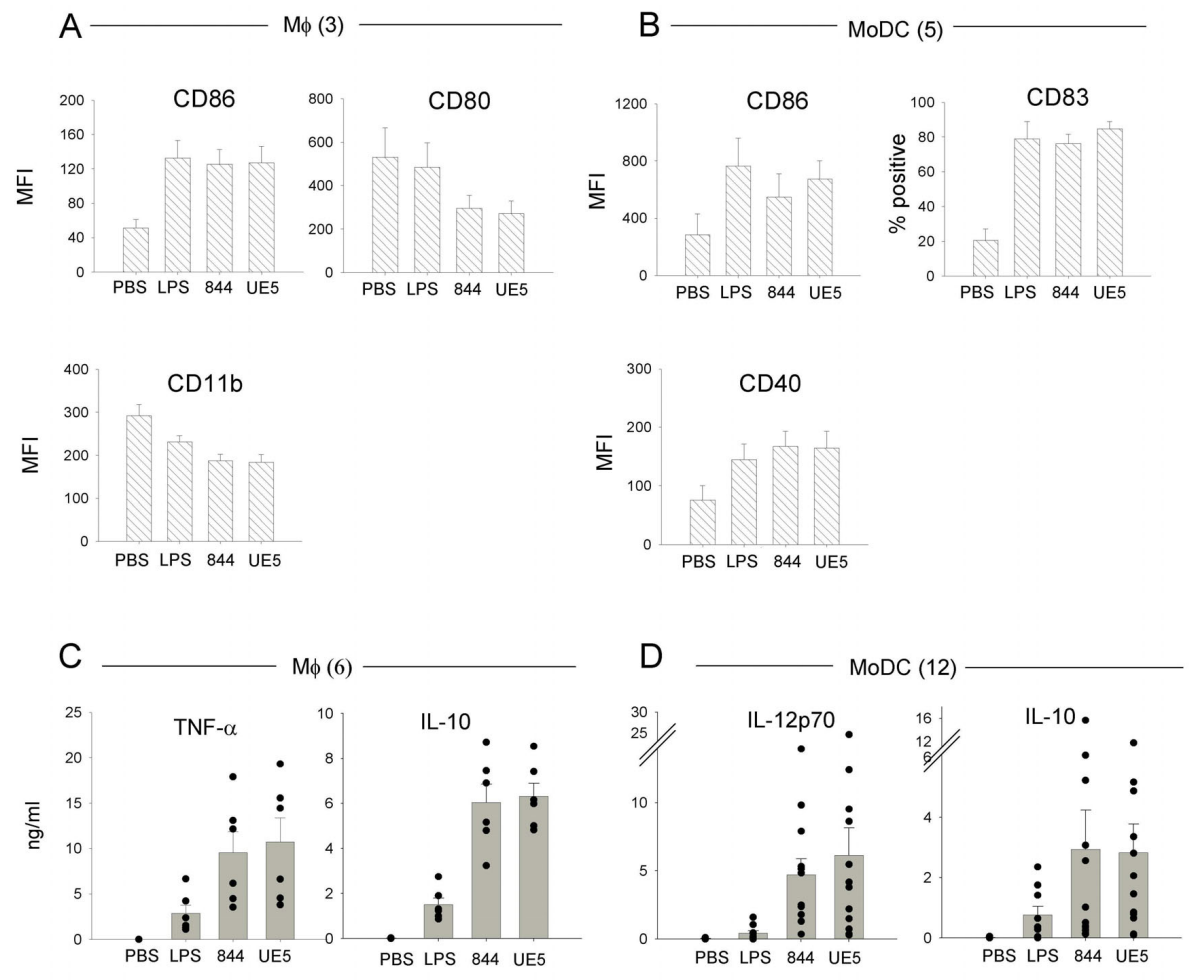
**Host cell responses upon *Bp*-844 and *Bt*-UE5 infection**. Mφs (A and C) and MoDCs (B and D) were infected with *Bp*-844 or *Bt*-UE5 at MOI of 1 for 24 hr and 250 μg/ml kanamycin was added 2 hr after infection. Expression levels of CD86, CD80 and CD11b of Mφs (A) and CD86, CD83 and CD40 of MoDCs (B) on the cell surface were determined with flow cytometry and the results are shown as mean and SEM. Cytokines in supernatants of infected Mφs (C) and infected MoDCs (D) were measured by ELISA and results are shown as mean and SEM, with each symbol representing the level from individual donor. *E. coli *LPS (1 μg/ml) and PBS were used as controls. Number in parenthesis indicates the number of donors for each experiment. MFI = mean fluorescence intensity.

Given the different fates on intracellular bacterial survival profiles noted above, it was of interest to examine the effect of bacterial infection on the production of host costimulatory molecules, surface markers and cytokines. Exposure of MoDCs to *Bp*-844 and *Bt-*UE5at a MOI of 1 for 24 hr readily induced cell maturation as judged by significant increases in the levels of the maturation marker, CD83 (Figure 2B). Under the same conditions, both *Bp*-844 and *Bt*-UE5 induced upregulation of the costimulatory molecule, CD86, in MoDCs and Mφs (Figure 2A and 2B). The effect of bacterial infection on the upregulation of other costimulatory molecules varied. For example, while the levels of CD40 (Figure 2B) and CD80 (data not shown) in MoDCs also increased, it was rather surprising to observe that infection of Mφs with either *Bp*-844 or *Bt*-UE5 actually caused a reduction in the levels of CD80 and CD11b (Figure 2A). The reduction noted with the Mφs is unlikely to be related to a technical artifact because when compared with the background MFI (mean fluorescence intensity) obtained with PBS, *Escherichia coli *LPS also slightly depressed CD11b expression, a finding that is consistent with earlier reports by other investigators using some other human Mφmodels [7,8].

Infection with either *Bp*-844 or *Bp*-UE5 stimulated similar degrees of cytokine secretion in MoDCs and Mφs. Significant elevations in the levels of IL-10 and TNF-α (*P *< 0.05, paired-samples *t*-test) were found in the supernatant fluid of the Mφcultures infected with either *Bp*-844 or *Bt*-UE5 (Figure 2C). Infection with either organism also stimulated production of IL-10 and IL-12p70 (*P *< 0.05, paired-samples *t*-test) in the MoDC cultures (Figure 2D). The level of IL-12p70 and not the TNF-α was determined in the MoDC supernatants because it is more readily related to the polarization of naïve T cells to Th1 phenotype [9]. The proportion of pro-inflammatory to anti-inflammatory cytokines induced by these 2 bacteria was calculated and found to be not different from one another (data not shown). It should be mentioned that the changes in the levels of costimulatory molecules and the increased secretion of cytokines induced by these 2 bacteria did not depend on their invasive properties or the numbers of bacteria internalized as exposure to organisms that had been killed by PFA treatment induced changes in cytokine and costimulatory molecule levels that were similar to those observed using living bacteria (data not shown). In summary, *Bp*-844 and *Bt*-UE5 appeared to exert roughly similar influences on the cellular responses of MoDCs and Mφs, judging from the expression levels of costimulatory molecules and cytokine production.

### T helper cell differentiation profiles induced by *Bp*-844- and *Bt*-UE5-stimulated MoDCs

**Figure 3:**
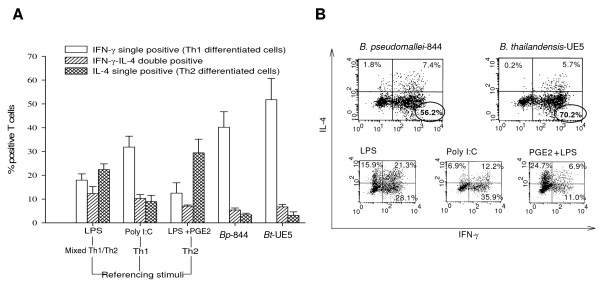
**T helper cell differentiation profiles driven by *Bp*-844- or *Bt*-UE5-stimulated MoDCs**. MoDCs were allowed to mature after priming with PFA-fixed *Bp*-844 or *Bt*-UE5 for 24 hr. These matured MoDCs were then co-cultured with allogeneic T cells for approximately 2 weeks as detailed in the Methods section. Afterward, the resting differentiated T cells were restimulated with PMA/ionomycin and their intracellular IL-4 and IFN-γ levels were analyzed by flow cytometry. *E. coli *LPS, poly I:C and *E.coli *LPS + PGE2 were used as referencing stimuli for mixed Th1/Th2, Th1 and Th2, respectively. Results (mean and SEM) of the T cell responses from 5 different MoDC donors interacting with a same batch of allogeneic T cells are shown as per cent of responsive positive T cells in A and a scatter plot from one representative donor is shown in B. Circles indicate the predominant Th1 cell population induced by *Bp*-844 and *Bt*-UE5.

We compared the ability of *Bp*-844-*and Bt*-UE5-stimulated MoDCs to induce Th1-Th2 differentiation. A MoDC-allogeneic naïve CD4^+ ^co-culture system was employed essentially as described earlier by Bergman *et al*. (9). MoDCs from 5 different donors that had been exposed to PFA-fixed *Bp*-844 or *Bt*-UE5 were incubated with allogeneic T cells and the extent of T-cell polarization was determined (Figure 3A). Scatter plots of flow cytometry analyses from one representative MoDC donor are separately shown in Figure 3B. The results with all 5 donors showed that MoDCs exposed to either of the PFA-fixed bacteria biased toward a Th1 polarization, based on the observation that the majority of T cells in samples that had been exposed to the bacteria produced intracellular IFN-γ which is a characteristic of Th1 cells. In conclusion, stimulation of MoDCs with either *Bp*-844 or *Bt*-UE5 induced indistinguishable T-helper cell profiles, i.e., both predominantly induced naïve T cells to differentiate to a Th1 cell population.

## Discussion

Different lines of evidence from patients with melioidosis, including the elevation of pro- and anti-inflammatory cytokines in serum, suggest the importance of innate immunity in determining the outcome of infection [2,10]. There is also evidence in humans showing that recovery from melioidosis may depend, in part, on cell-mediated arm of adaptive immune system [11]. The latter is partially dependent on the type of Th cell differentiation which is controlled in part by activated DCs. Studies of experimentally induced melioidosis in different inbred strains of animals have shown the importance of genetic background in determining the course of the infection [12]. Despite numerous studies in animal melioidosis models using mutant and wild type bacteria [13,14] a complete understanding of the mechanisms involved in pathogenesis, particularly in humans, has yet to be realized. We felt that additional approaches looking at the effects of infection on isolated human phagocytic cell populations would contribute new insights into the pathogenic mechanism of the disease. We also reasoned that using virulent *Bp *as well as its genetically related avirulent natural counterpart, *Bt*, might highlight aspects of the host response that play a role in virulence and pathogenesis. Moreover, the use of primary human cells, instead of animal cells or cell lines, would yield results that are more relevant in regard to the human disease. We had previously demonstrated that this same *Bp *strain adhered to and invaded a non-phagocytic human lung alveolar cell line more effectively than *Bt*-UE5 [6]. In the present study, we continued this same approach, but this time using primary human phagocytic cells instead of a non-phagocytic human and animal cell lines. After internalization, we found that *Bt *strain UE5 but not *Bp *strain 844, had a reduced survival rate and failed to multiply in the Mφs (Figure 1A). We have limited data confirming that another *Bp *stain (1026b) could also survive and readily replicate inside primary human macrophages (unpublished). From the current information regarding the different characteristics between these 2 *Burkholderia *species, we suspect that exopolysaccharide capsular components [13] or the type-III secretion system cluster 1 [15], which are absent in *Bt*, may be responsible for the differences in their intracellular survival kinetics. Currently, we have data showing that, compared with its wild type counterpart, a capsule mutant of *Bp *that lacks the exopolysaccharide component was at least 10^5^-fold less virulent in BALB/c mice and it also survived and replicated poorly inside primary human Mφs (manuscript submitted).

Furthermore, our results showed that following IFN-γ activation, both MoDCs and Mφs acquired an enhanced ability to kill *Bp*-844. The significance of IFN-γ in conferring resistance against experimental melioidosis has been reported previously by several groups of investigators [16,17] including our own group [18]. Unactivated MoDCs, while possessing an antigen-presenting capacity for the stimulation of the adaptive immune response that is superior to that of Mφs, were unable to inhibit the intracellular growth of either *Burkholderia *strains (Figure 1B). However, after IFN-γ pre-activation, an enhanced intracellular killing capacity was observed in the MoDCs infected with *Bp*-844 (Figure 1D). *In vivo*, IFN-γ can be produced by NK cells or Th1 cells [16], but their arrival at the initial site of infection might be too late to provide an effective antibacterial response. We previously showed that injecting BALB/c mice with CpG ODN to enhance IFN-γ production and innate immunity in general could effectively protect the animals against a subsequent challenge with virulent *Bp *[18]. Furthermore, we found that if the CpG ODN administration was given closer to the time of bacterial challenge, the degree of protection was also reduced. As demonstrated in the present study, the enhanced *in vitro *killing of Bp-844 by human MoDCs and Mφs pre-stimulated with IFN-γ (Figure 1C) is in accord with the previously mentioned *in vivo *observations in experimental animals. Taken together, it appears that the host innate immune response is able to distinguish *Bp*-844 from *Bt*-UE5 (Figure 1), and this may in turn influence the outcome of primary infection by *Bp*. Once the infection has progressed beyond this stage, the host adaptive immune system will be activated but may not be able to distinguish between these 2 bacteria, as demonstrated by our results showing the similar patterns of costimulatory molecule expression, cytokine production and strong bias toward a Th1 polarization (Figures 2 and 3). In addition, the results of our *in vitro *studies showing that human MoDCs that have been exposed to *Bp*-844 have predominant biased effects on Th1 cell differentiation (Figure 3) are consistent with the results of a previous study showing strong Th1 cell-mediated immune responses in melioidosis patients who had recovered from melioidosis [11]. Moreover, a mixed Th1/Th2 response has also been reported in animal studies [12]. The detection of some activated T cells exhibiting a mixed Th1/Th2 response producing both intracellular IFN-γ and IL-4 in this study (Figure 3) is in accord with the animal study just mentioned. Further investigations using the human phagocytic cell system described in the present study are needed if one is to more fully understand the pathogenesis of human melioidosis. We are currently investigating the possible role and involvement of polymorphonuclear cells in the disease process in humans.

## Conclusion

Our comparative study showed that while both *Bp*-844 and *Bt*-UE5 were similar in their ability to survive and replicate in human MoDCs, only *Bp*-844 could replicate in human Mφs. Both bacteria, however, possessed a similar capacity to stimulate DC maturation, cytokine production and T cell polarization toward a Th1 phenotype. It is possible that the superior ability of *Bp*-844 to survive and replicate in phagocytes may contribute to the virulence of the organism and thus may influence the outcome of infection in the human host.

## Methods

### Bacterial strains

The clinical isolate, *Bp *strain 844, and the non-pathogenic environmental isolate, *Bt *strain UE5, were used in this study. These strains have been used and more fully characterized in our previous reports [3,6,19]. In brief, *Bp*-844 is a clinical isolate from a patient admitted to a general hospital in the endemic Khon Kaen province in Thailand. It possesses an exopolysaccharide capsular component, can form biofilms and has a typical LPS phenotype. The environmental soil isolate, *Bt*-UE5, while it does not possess an exopolysaccharide capsule, exhibits a similar LPS phenotype and biofilm component. Doubling times in a cell-free system for both strains were calculated and found to be very similar: 40 min for *Bp*-844 and 37 min for *Bt*-UE5. Late log phase cultures of *Bp*-844 and *Bt*-UE5 were achieved by inoculating them in trypticase soy broth for 16 hr in a rotating incubator. The number of bacteria was estimated according to the culture's optical density value at 650 nm and the exact number of colony-forming unit (cfu) was subsequently determined by serial dilution, plating on trypticase soy agar and colony counting [3]. Paraformaldehyde (PFA)-fixed bacteria used in MoDC-driven T helper cell differentiation were prepared by treating viable bacteria with 1% PFA for 15 min.

### Primary human MoDC and Mφ cultures

Blood was collected from healthy adult volunteers who lived in non-endemic areas for melioidosis in Thailand. They were considered healthy based on their physical appearance together with past medical history and were currently free from any illness and had not taken pharmaceutical agents at least one week prior to the time of blood withdrawal. Ethical clearance for blood collection from donors was approved by the Ethical Clearance Committee of Ramathibodi Hospital, Mahidol University, Bangkok, Thailand (Ethical clearance number 2549/452). Peripheral blood mononuclear cells were prepared using Ficoll-Hypaque separation method [20] and CD14^+ ^monocytes were isolated using a magnetic cell sorter system as recommended by the manufacturor (MACS^®^, Miltenyi Biotech GmbH, Bergisch Gladbach, Germany). Immature MoDCs were prepared and cultured as described [21] except that they were cultured in the absence of antibiotics. Briefly, the purified CD14^+ ^monocytes were cultured for 5–6 days in 5% CO_2_, 37°C in RPMI 1640 (Gibco, Grand Island, NY) supplemented with 10% fetal calf serum (BioWithaker, Cambrix, UK), 2 mM L-glutamine, 1% non-essential amino acids, 1 mM sodium pyruvate and 0.05 mM 2-mercaptoethanol, in the presence of 100 ng/ml IL-4 and 100 ng/ml GM-CSF (both from R&D Systems, Minneapolis, MN). The cells generated as such exhibited morphological and surface characteristics typical of dendritic cells. Unstimulated Mφs were obtained by culturing the purified CD14^+ ^monocytes in 10% inactivated human AB serum (GemCell™, West Sacramento, CA) supplemented with 1% glutamine in ISCOV's MDM (Gibco) for 5–6 days before used in the experiment [22].

### Determination of survival and growth kinetics of bacteria inside MoDCs and Mφs

The number of intracellular bacteria was determined using an antibiotic protection assay [16]. In brief, host cell monolayers were exposed (T_0_) to viable *Bp*-844 or *Bt*-UE5 at a multiplication of infection of 1 for 2 hr before extracellular bacteria were eliminated by treating the cells with 250 μg/ml kanamycin for 1 hr (T_3_). Thereafter, the cells were cultured in medium containing 20 μg/ml kanamycin to suppress growth of any residual viable bacteria that might still be present. At the time points indicated, the host cells were lyzed by treating with 0.1% Triton X-100 for 5 min and the number of intracellular bacteria were counted using standard plate pouring technique [3]. In the case of IFN-γ pre-stimulation, host cell monolayers were treated with recombinant human IFN-γ (CytoLab Ltd., Rehovot, Israel) for 16 hr before the infection was performed. Host cell viability was determined using 0.04% trypan blue staining to ensure that more than 90% of the cells remained alive at the final time point.

### Determination of surface marker expression and cytokine production by infected cells

MoDCs or Mφs were infected with viable *Bp*-844 or *Bt*-UE5 at a MOI of 1 for 2 hr before 250 μg/ml of kanamycin was added. Twenty-two hr later, supernatants were collected for cytokine assay while infected host cells were dislodged for determining the surface expression of CD40, CD80, CD83, CD86 and CD11b upon incubation with fluorescently-labelled antibodies (BD Biosciences, San Jose, CA) and analyzed by flow cytometry (FACSCanto, BD Biosciences) as described [7,9,21]. Levels of cytokines in supernatants were measured using commercial ELISA kits: IL-12p70 and IL-10 (both from BD Biosciences) and TNF-α (R&D Systems).

### Determination of DC-driven Th1-Th2 differentiation

MoDCs were stimulated for 24 hr with PFA-fixed *Bp*-844 or *Bt*-UE5 at a MOI of 1 and their ability to induce allogeneic T cell differentiation was determined [9]. In brief, *Bp*-844- or *Bt*-UE5-activated MoDCs were co-cultured with allogeneic CD45RA^+^CD4^+^T cells at a ratio of 5,000 MoDCs per 20,000 T cells for 12–14 days in the presence of 100 iU/ml IL-2 (R&D Systems) from day 5 onwards. Differentiated T cells were then restimulated with 10 ng/ml PMA and 1 μg/ml inonomycin (both from Sigma-Aldrich, St. Louis, MO) for 6 hr. Intracellular IFN-γ and IL-4 production were measured by flow cytometry on a single cell basis and the type of T cell differentiation was determined based on the ratio of IFN-γ and IL-4 producing cells. Each set of experiments always included control agents referencing for different T cell polarization, i.e., 5 μg/ml *E. coli *LPS (Sigma-Aldrich) for mixed Th1/Th2, 100 μg/ml poly I:C (Invitrogen, Carlsbad, CA) for Th1 and 5 μg/ml *E. coli *LPS plus 10 μg/ml prostagrandin E2 (PGE2, Invitrogen) for Th2 [9].

### Statistical analysis

Differences between 2 groups were analyzed using paired-samples *t*-test and differences among multiple groups were analyzed using the one-way ANOVA test. *P*-values of < 0.05 were considered statistically significant.

## Abbreviations

*Bp*: *Burkholderia pseudomallei*; *Bt*: *Burkholderia thailandensis*; Mφs: macrophages; MoDCs: monocyte-derived dendritic cells; PFA: paraformaldehyde; LPS: lipopolysaccharide; PBS: phosphate buffered saline pH 7.4; PGE2: prostagrandin E2; cfu: colony-forming unit; MOI: multiplicity of infection.

## Authors' contributions

JC carried out the experimental procedures, performed the data analysis and drafted the manuscript. PU, AE and SS participated in the study design and helped to draft the manuscript. All authors read and approved the final manuscript.
